# Unveiling Human Values: Analyzing Emotions behind Arguments

**DOI:** 10.3390/e26040327

**Published:** 2024-04-12

**Authors:** Amir Reza Jafari, Praboda Rajapaksha, Reza Farahbakhsh, Guanlin Li, Noel Crespi

**Affiliations:** 1 Samovar, Telecom SudParis, Institut Polytechnique de Paris, 91120 Palaiseau, France; prr16@aber.ac.uk (P.R.); reza.farahbakhsh@telecom-sudparis.eu (R.F.); guanlin_li@telecom-sudparis.eu (G.L.); noel.crespi@telecom-sudparis.eu (N.C.); 2 Department of Computer Science, Aberystwyth University, Aberystwyth SY23 3DB, Ceredigion, UK

**Keywords:** human values, emotion analysis, language models, LLMs, GenAI

## Abstract

Detecting the underlying human values within arguments is essential across various domains, ranging from social sciences to recent computational approaches. Identifying these values remains a significant challenge due to their vast numbers and implicit usage in discourse. This study explores the potential of emotion analysis as a key feature in improving the detection of human values and information extraction from this field. It aims to gain insights into human behavior by applying intensive analyses of different levels of human values. Additionally, we conduct experiments that integrate extracted emotion features to improve human value detection tasks. This approach holds the potential to provide fresh insights into the complex interactions between emotions and values within discussions, offering a deeper understanding of human behavior and decision making. Uncovering these emotions is crucial for comprehending the characteristics that underlie various values through data-driven analyses. Our experiment results show improvement in the performance of human value detection tasks in many categories.

## 1. Introduction

Understanding human values is a multifaceted endeavor that lies at the intersection of psychology, sociology, and philosophy. Values are the fundamental beliefs that guide individuals’ behaviors, decisions, and perceptions of the world around them. While values are often expressed through rational arguments and logical reasoning, they are also deeply intertwined with emotions. Emotions play a crucial role in shaping our values, influencing the way we perceive events, interact with others, and form opinions.

Analyzing fine-grained emotions from text is a significant part of extracting information from different texts, and utilizing emotion features can express more subtle and complex sentiments and improve detection performance in various NLP tasks, such as modeling and emotion recognition in human behavior [1]. Unravelling these emotions becomes vital for understanding the characteristics behind different values through data-driven analyses. Fine-grained emotion analysis enables us to not only identify the overarching emotions present in these interactions but also to distinguish between subtle emotional nuances. For instance, it can differentiate between the anger fueled by perceived injustice and the excitement of discovering a new perspective. Such distinctions are crucial for uncovering the motivations and thought processes that underlie individuals’ value systems.

As an extension of our previous study [2], we aim to explore the complex relationship between human values and emotions by focusing on the analysis of emotions behind arguments. We recognize that arguments, whether they occur in everyday conversations, political debates, or scholarly discourse, are not solely driven by logical reasoning but are also profoundly influenced by the emotional factors that accompany them. By examining the emotional aspects of arguments, we aim to uncover the emotional features underlying different levels of human values that shape and inform these exchanges. In our analysis, we extend beyond sentiment analysis as it provides a limited scope in capturing the complexity of human emotions. Fine-grained emotion analysis, on the other hand, provides a finer view, focusing on a wide range of emotions such as joy, sadness, surprise, fear, and disgust [3]. By incorporating this level of granularity into our analysis, we can gain a deeper understanding of how specific emotions influence the formation and evolution of human values.

As we delve deeper into this research, we investigate not only identifying emotions but also exploring the relationships between these emotions and different levels of human values. We seek to explore how emotions manifest in arguments and what they reveal about the values held by individuals and societies. Ultimately, we aim to conduct an experiment to utilize the extracted emotional features from arguments to improve the performance of the human value detection task.

The remainder of the paper is organized as follows: Section 2 presents an overview of the background knowledge and related works on human value detection and emotion analysis. Section 3 delves into the analysis of the emotional dimensions associated with human value levels. Our experimental results and findings are discussed in detail in Section 4. Finally, Section 5 concludes our work and outlines potential future directions for research in this field.

## 2. Background and Related Works

Human values are the fundamental beliefs and principles that guide individuals’ behavior, decisions, and interactions with others. These values are deeply embedded within cultures and societies and serve as guiding principles for ethical behavior, shaping individuals’ attitudes, priorities, and actions. They play a crucial role in shaping personal identity, social cohesion, and the overall fabric of society. Based on Schwartz’s theory [4], there are basic values that are universally recognized across cultures such as Self-Enhancement, Self-Transcendence, Openness to Change, and Conservation. These values are structured in a circular pattern, with opposing values positioned opposite each other on the circle. Individuals and cultures may prioritize certain values over others, leading to variations in behavior, attitudes, and societal norms [5,6,7,8].

**Figure 1:**
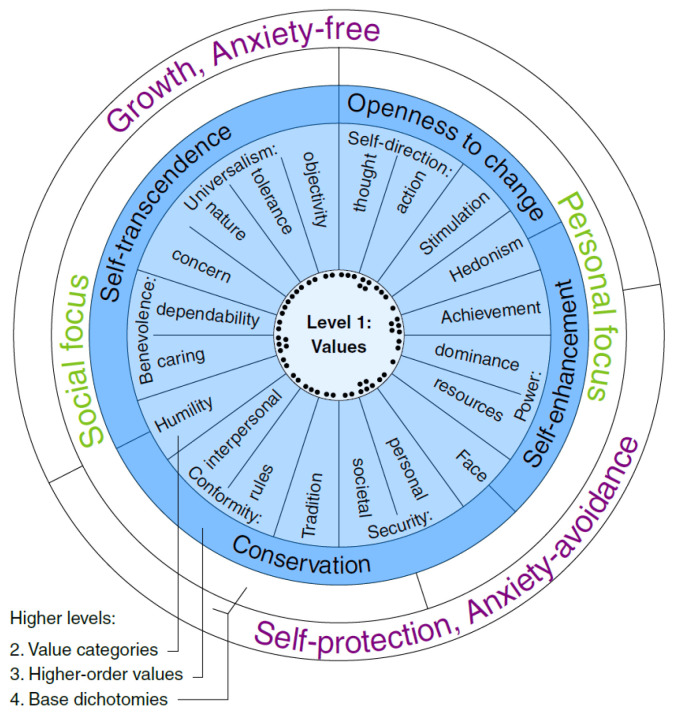
Illustration of the taxonomy of human values and their hierarchical levels (1–4), selected for their relevance in social science research [10]. Level 1 is associated with 54 values, which are classified into the more abstract categories found at levels 2–4.

Human arguments are ways people express and confirm their values using different styles, words, and goals. With digital communication being so widespread nowadays [9], Kiesel et al. [10] introduced a comprehensive multi-level taxonomy of human values considering natural language arguments, comprising 54 distinct values categorized on four levels, as depicted in Figure 1. However, the task of detecting human values via machine learning algorithms is challenging due to the sheer diversity of values and their often implicit presence within arguments. In this regard, a dataset has been introduced in task 4 of the SemEval 2023 for Identifying Human Values behind Arguments (https://touche.webis.de/clef24/touche24-web/human-value-detection.html accessed on 28 March 2024) that incorporates over 9000 arguments mapped to a consolidated multi-level taxonomy [11]. Consequently, recent research efforts have been dedicated to enhancing the effectiveness of such detection methodologies that contain a variety of approaches aimed at refining the performance of algorithms tasked with identifying and categorizing human values within the discourse landscape [12,13]. For example, from an architectural perspective, Schroter et al. reached the best-performing approach by ensembling transformer-based models [14], and Hemati et al. proposed a two-stage pipeline architecture called Label Graph Transformer to find the interactions between labels [15]. In [16], the authors proposed a similarity-aware model by taking a similarity score approach.

Emotions and sentiments play a major role in extracting knowledge from different sources. Therefore, the tasks of emotion detection and sentiment analysis have gained significant attention in recent years [17,18]. Leveraging emotion features extracted from various sources has proven to be beneficial in enhancing performance across a multitude of domain-specific tasks. For example, Bandhakavi et al. [19] harnessed weakly labelled data from blogs, news headlines, and tweets to extract effective features for emotion classification. In the question-answering task, Gui et al. [20] introduced a novel approach that extracts both word-level sequence features and lexical features to improve emotion cause extraction. Furthermore, fine-grained emotion features have demonstrated their efficacy in enhancing the performance of implicit hate speech detection within a multi-task setup alongside sentiment analysis [21].

To the best of our knowledge, previous studies have not explored the utilization of emotion features in human value detection tasks. Therefore, following a thorough review and extraction of emotions associated with various levels of human values, we aim to leverage these features to enhance the effectiveness of human value detection by extending our previous study [2].

## 3. Emotion Analysis of Human Values

In this study, we analyzed fine-grained emotions utilizing the three taxonomies provided by the GoEmotions model [22]. The original taxonomy of this model comprised 27 emotions including the neutral category, which is a finer-grained version of two other taxonomies: Hierarchical Grouping (positive, negative, ambiguous) and Ekman’s six main emotion categories (anger, disgust, fear, joy, sadness, surprise) including neutral. However, we ignored the ‘Neutral’ label from our analysis to focus more on the conflicting emotion combinations in our analysis.

To extract emotion knowledge from arguments, we utilized a human value dataset containing 9324 arguments in total [11]. The human value dataset was generated based on the taxonomy with four different levels (Figure 1).

- **Level 1** is associated with 54 fine-grained values, sourced from cross-cultural social science studies. Each value encapsulates a widely accepted belief that aligns with the principles of psychology. While some values such as ‘Have a stable society’ or ‘Be responsible’ are commonly accepted among different people and societies, others such as ‘Be holding religious faith’ or ‘Have freedom of action’ may provoke more controversy owing to variations in priorities among individuals and cultures.
- **Level 2** encounters value categories, which contain 20 distinct categories associated with the 54 values. These categories span a broad spectrum, including facets like Self-direction, Universalism, and Benevolence, offering a comprehensive framework for understanding human values across different dimensions.
- **Level 3** introduces higher-order value conflicts, drawing upon Schwartz’s work [4]. This level explores conflicts such as openness to change versus conservation and self-transcendence versus self-enhancement, shedding light on the intricate dynamics between contrasting sets of values.
- **Level 4** comprises two sublevels. Level 4a focuses on different personal and social focus, considering the divergent motivational tendencies underlying individual and collective value systems. Meanwhile, Level 4b further divides into Self-protection, Anxiety-avoidance, and Growth, Anxiety-free, offering insights into the underlying motivations that drive human behavior in pursuit of value fulfilment.

We employed the Huggingface implementation of the GoEmotion model (https://github.com/monologg/GoEmotions-pytorch accessed on 28 March 2024) to quantify emotions associated with each value category. This Transformer-based model computes a probabilistic score for each emotion, ranging from 0 to 1, where the higher score for the output emotion shows a higher possibility of containing that emotion, offering insights into the likelihood of its occurrence. For our experiments, after testing different thresholds for the model, we set the threshold at 0.1 to encompass a broad spectrum of emotions in the output model for each value.

**Figure 2:**
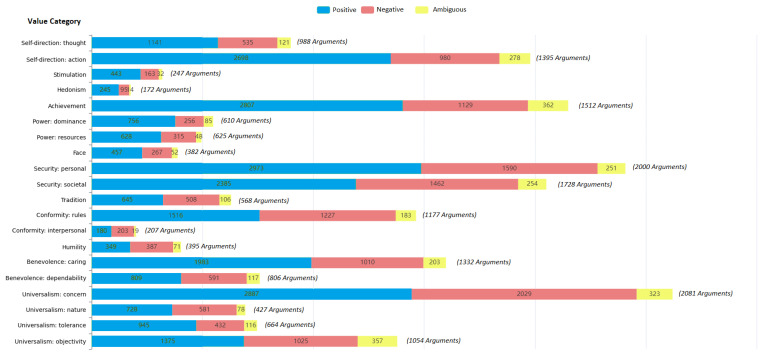
The number of extracted emotions from each human value category (Level 2). (The total number of arguments corresponding to the value category is shown in front of each category). It shows the bias distribution of arguments in each category; however, there is at least one emotion assigned to the argument.

Using the above taxonomies and datasets, we explored the distribution of extracted emotions across three emotion levels (positive, negative, ambiguous) within each human value category (level 2). This analysis primarily focused on level 2 emotion analysis, which serves as the foundation for evaluating both baseline and proposed models in Section 4. Figure 2 illustrates the visual representation of the emotional landscape associated with different values. This distribution shows that while there is a bias in the number of arguments in each human value category, each argument contains more than one emotion label, especially positive emotions.

### 3.1. Levels 1 and 3 Analysis

**Figure 3:**
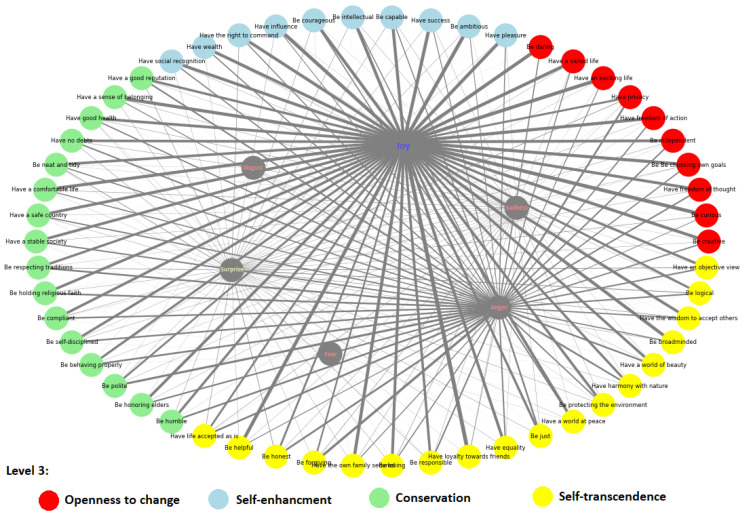
Network model of Ekman emotion label (in gray color nodes) with target human values at level 1. Colored nodes represent the corresponding level 3 human value labels and edge size based on the label frequency of the emotion assigned to the value. It shows the overall tendency of ‘joy’ (as positive) and ‘anger’ (as negative) emotions but with different distributions at level 3.

We initiated our emotion analysis focusing on the 54 human values at level 1 of the taxonomy, along with their corresponding higher-order human values at level 3, employing the Ekman setup. As depicted in Figure 3, our findings suggest that ‘joy’ is a prevalent positive emotion linked to these human values with the ‘Have freedom of Action’ as the strongest associated human value while ‘anger’ stands out as a dominant negative emotion category. We observed that the rest of the negative emotional categories, namely disgust, fear, and sadness, rank notably lower across nearly all human values. However, ‘surprise’ exhibits a relatively uniform distribution across various human value categories.

The level 1 analysis helped us to understand how emotions generally spread among all values and how individuals emotionally engage with and respond to different values.

Upon taking a broader perspective at the higher-order level of human values, we observe distinct patterns within the contrasting value categories. Specifically, a detailed examination of Figure 3 reveals that values such as ‘Be polite’, ‘Be respectful of traditions’, and ‘Be humble’, all fall under the ‘Conservation’ category, exhibiting a more balanced distribution of emotions compared to values categorized under ‘Openness to Change’, which tend to lean towards positive emotions.

Indeed, the same pattern is not as explicitly observed when contrasting the categories of ‘self-transcendence’ and ‘self-enhancement’ concerning positive emotions. Conversely, when examining negative emotions, ‘self-enhancement’ values exhibit a relatively lower overall percentage compared to ‘self-transcendence’ values.

These contradictions offer valuable insights that can be used to enhance the performance of human value detection in our experiments. By leveraging these nuances, we can refine our methodologies to better discern and interpret the intricate connections between emotions and human values.

### 3.2. Level 2 Analysis

**Figure 4:**
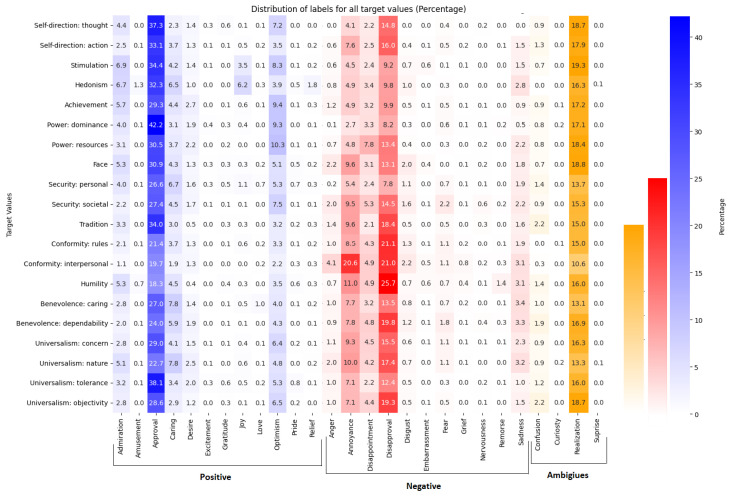
Fine-grained emotion distribution of human values. The y-axis represents the value category of the dataset at level 2, and the x-axis shows the fine-grained emotions from the original taxonomy of GoEmotions. The blue color in this heatmap is assigned for emotions under positive sentiment categories, and red and orange indicate emotions of negative and ambiguous categories, respectively, [2].

Transitioning to level 2 of our human values analysis, we utilized the original configuration of the emotion model with 27 emotions to obtain a broader perspective of various emotion distributions. Our analysis focused on fine-grained emotions, consisting of 27 emotions categorized into three main groups: positive, negative, and ambiguous. As depicted in Figure 4, in positive emotions, most human values align with the ‘Approval’ category followed by ‘optimism’, ‘admiration’, and ‘caring’ as the next favored positive emotions. The other positive emotions contain a small portion of overall distributions. Considering negative emotions, ‘disapproval’ and ‘annoyance’ emerge as dominant categories.

This observation suggests that during arguments centered on negative opinions, individuals often aim to express disapproval of certain values or experiences that evoke annoyance, while positive opinions tend to convey approval of specific values. Furthermore, the prevalence of ‘realization’ among ambiguous emotions indicates that these values do not strongly lean towards either approval or disapproval. Notably, these emotional nuances hold significant potential as additional features for enhancing the classification process.

Moreover, given that the dataset comprises arguments structured with a premise, a conclusion, and a stance indicator specifying whether the premise supports or opposes the conclusion, it is noteworthy that the extracted emotions predominantly reflect opposing sentiments, such as ‘approval’ and ‘disapproval’.

In conclusion, the extracted information from emotion distribution in all human value levels highlights the significance of particular emotions with varying polarities in human value arguments, suggesting their potential as valuable features for enhancing the performance of human value detection algorithms which are presented in the next section.

## 4. Results and Discussion

We experimented to assess the influence of utilizing emotion features in the human value classification for the detection of human values at level 2 (to compare the performance with the reported results in the base paper [11]). First, we acquired the emotion features from the argument dataset and then we passed these features to the main BERT model [23]. By augmenting the extracted emotion features with textual features, we generated the results presented in Table 1. These findings highlight the considerable advantages of incorporating emotion-related data into assessing human values. Our model (BERT + emotions) consistently surpassed the 1-Baseline across all evaluated categories. Notably, compared to the primary BERT model, we achieved a 2% increase in micro-average performance, with particularly substantial improvements observed in categories such as ‘Stimulation’, ‘Face’, ‘Humility’, and ‘Benevolence: dependability’. Overall, our model performed better in 12 categories out of 20 and is equal to the base BERT model in 2 categories.

In evaluating our model’s performance, we selected three distinct categories that showed different patterns in performance. Firstly, in the ‘Stimulation’ category comprising 247 arguments, our model demonstrated significant improvements compared to both the baseline and the primary BERT model, achieving an accuracy of 0.24, surpassing the respective scores of 0.09 and 0.05.

Secondly, in the ‘Conformity: interpersonal’ category, which consisted of 207 arguments, our model did not exhibit any improvement compared to the base BERT model, yielding an accuracy of 0.23.

Lastly, in the ‘Universalism: nature’ category encompassing 427 arguments, while our model managed to outperform the baseline, it fell short of surpassing the performance of the primary BERT model (0.62 vs. 0.71).

To unravel the influence of emotional features within these three selected categories, we delved into 27 fine-grained emotions, selecting two positive categories (approval and optimism), two negative categories (annoyance and disapproval), and one ambiguous category (realization) as the dominant emotion categories among all.

**Figure 5:**
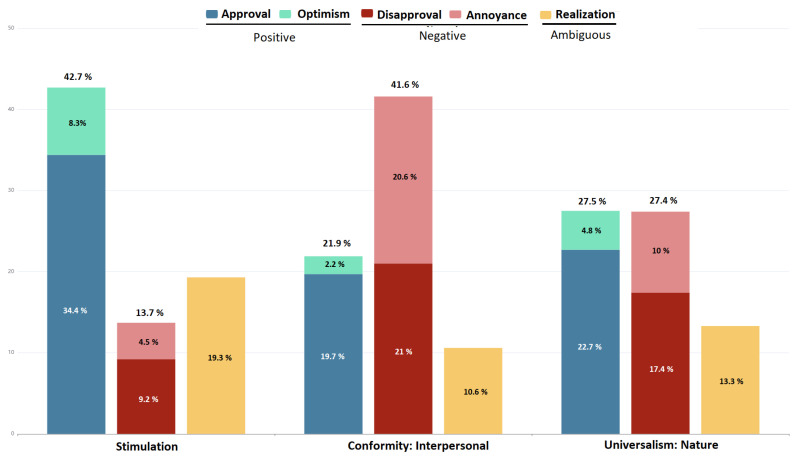
The distribution of dominant emotion categories for the three selected human value categories where our model outperformed both the baseline and BERT (Stimulation), where there was no improvement compared to BERT (Conformity: interpersonal), and where it failed to reach base BERT performance (Universalism: nature).

As depicted in Figure 5, our model exhibited its strongest performance when the distribution of positive emotions (approval and optimism) outweighed the negative emotions (42.7% for positive vs. 13.7% for negative of overall emotion distribution). Conversely, in categories where negative emotions dominated, such as ‘Conformity: interpersonal’, with a prevalence of 41.6%, our model only marginally outperformed the baseline, with emotion features failing to enhance the performance of the main BERT model. Interestingly, when confronted with an even distribution of positive and negative emotions, our model struggled to match the performance of the primary BERT model. This disparity could potentially stem from a lack of bias in the emotion distribution.

While these overarching patterns shed light on the interplay between emotion distribution and model performance, it is crucial to acknowledge that other variables may also contribute to the poor performance of the model in some categories. For instance, the limited number of arguments for various human value categories could hinder the model’s ability to effectively generalize across diverse datasets. Exploring these additional factors will be instrumental in further refining our understanding and improving the performance of our models in human value detection tasks.

To gain a better understanding of the results gained by our model and the relation between various emotions with human value categories, we employed the Pearson correlation coefficient, a robust statistical method widely recognized for evaluating linear relationships between variables. To comprehensively analyze the spectrum of emotions in human values, we conducted correlation analyses specifically between Hierarchical Grouping emotions (positive, negative, ambiguous) in levels 3 and 4 of human values, since these two levels show contradicting perspectives from human values and may be a good perspective from which to analyze the reason for lower performances in related categories. The Pearson correlation is calculated as follows, where $x_{i}$ and $y_{i}$ represent individual data points and $\bar{x}$ and $\bar{y}$ are their respective means. The correlation coefficient (r) ranges from −1 to 1, where 1 indicates a perfect positive and linear relationship, −1 indicates a perfect negative linear relationship, and 0 indicates no linear relationship between emotion labels in human value categories.

$$r=\frac{\sum_{i=1}^{n}\left(x_{i}-\bar{x}\right)\left(y_{i}-\bar{y}\right)}{\sqrt{\sum_{i=1}^{n}{\left(x_{i}-\bar{x}\right)}^{2}}\sqrt{\sum_{i=1}^{n}{\left(y_{i}-\bar{y}\right)}^{2}}}$$

The correlation analysis conducted on levels 3 and 4 of human values provides insightful findings, as presented in Table 2 and Table 3. Across all emotion labels, we consistently observe high levels of correlation between emotion categories. However, our emotion model generally fell short of outperforming the base BERT model in most categories, falling under the ‘Self-transcendence’ category at level 3. Correspondingly, the correlation results for this category also exhibit lower values compared to other categories.

In contrast, our model exhibited superior performance in all subcategories of ‘Self-enhancement’, surpassing both the baseline and the main BERT model, with the correlation values also reaching the highest levels for this category.

Taking a closer look at Table 2, when comparing the Social Focus category with the Personal Focus category, while the output for negative and positive emotions appears quite similar, notable differences emerge in the ambiguous and positive emotions, with correlations of 0.8266 for Social Focus and 0.9648 for Personal Focus.

Within the Self-protection and Anxiety-avoidance category, the correlation results consistently exceed 0.95 for all emotions, indicating strong associations. Conversely, in the Growth and Anxiety-free category, correlations do not surpass 0.88. This variance in emotion distribution patterns among related values in this category may contribute to the observed discrepancy.

## 5. Conclusions and Future Works

In the initial stages of our comprehensive analysis, we illuminated the diverse distribution of emotions across various human value categories, laying the foundation for the integration of these features into our classification model. This insightful exploration prompted the innovative idea of harnessing emotion-related information to enrich the classification process. By incorporating these emotion features, we not only deepen our understanding of human values but also enhance the effectiveness of the classification model, underscoring the potential for significant advancements in this domain. Our analysis revealed diverse emotional patterns, including using contradictory emotions such as ‘approval’ and ‘disapproval’ within various human value categories. Additionally, via a correlation analysis, we unveiled the connections between emotions within each human value category. Ultimately, by incorporating emotion features into our experimental setup, we demonstrated an enhanced performance in detecting human values compared to the baseline method.

In our future research, we plan to build upon our findings by constructing a comprehensive knowledge graph using insights from emotional features and exploring additional methods for extracting information related to human values. Additionally, we tend to extract more complex relationships between emotion and human values by utilizing structural equation modeling. We also aim to leverage large language models (LLMs) and innovative techniques like chain-of-thought (CoT) prompting to enhance our reasoning capabilities. This integrated approach, combining emotion-driven analysis, knowledge graph development, and LLM-based reasoning, represents our strategic vision for advancing human value detection tasks through interdisciplinary analysis. 

## Figures and Tables

**Table 1:** Experimental result of human value classification (level 2) using emotion features and comparison with 1-Baseline and the main BERT model.

	BERT + Emotions			1-Baseline	BERT
**Value Category**	**Precision**	**Recall**	**F1-Score**	**F1-Score**	
Self-direction: thought	0.33	0.64	**0.44**	0.17	**0.44**
Self-direction: action	0.42	0.75	0.54	0.40	**0.55**
Stimulation	0.16	0.52	**0.24**	0.09	0.05
Hedonism	0.21	0.84	**0.34**	0.03	0.20
Achievement	0.47	0.73	**0.57**	0.41	0.56
Power: dominance	0.20	0.57	**0.30**	0.13	0.29
Power: resources	0.39	0.77	**0.52**	0.12	0.44
Face	0.19	0.55	**0.28**	0.12	0.13
Security: personal	0.62	0.89	0.73	0.51	**0.74**
Security: societal	0.45	0.88	**0.60**	0.40	0.59
Tradition	0.35	0.45	0.39	0.19	**0.43**
Conformity: rules	0.38	0.77	**0.51**	0.31	0.47
Conformity: interpersonal	0.19	0.30	**0.23**	0.07	**0.23**
Humility	0.20	0.44	**0.28**	0.09	0.07
Benevolence: caring	0.41	0.88	**0.56**	0.35	0.46
Benevolence: dependability	0.33	0.66	**0.44**	0.19	0.14
Universalism: concern	0.49	0.93	0.64	0.54	**0.67**
Universalism: nature	0.49	0.86	0.62	0.17	**0.71**
Universalism: tolerance	0.17	0.44	0.25	0.22	**0.32**
Universalism: objectivity	0.39	0.65	**0.49**	0.46	0.33
macro avg	0.32	0.68	**0.44**	0.26	0.42

**Table 2:** Pearson correlations among emotions in level 3 (Openness to change vs. Conservation) and (Self-transcendence vs. Self-enhancement).

	**Openness to Change**			**Conservation**		
	Positive	Negative	Ambiguous	Positive	Negative	Ambiguous
Positive	-	0.8746	0.8626	-	0.9779	0.9732
Negative	0.8746	-	0.8931	0.9779	-	0.9890
Ambiguous	0.8626	0.8931	-	0.9732	0.9890	-
	**Self-transcendence**			**Self-enhancement**		
	Positive	Negative	Ambiguous	Positive	Negative	Ambiguous
Positive	-	0.9498	0.7106	-	0.9919	0.9975
Negative	0.9498	-	0.7685	0.9919	-	0.9882
Ambiguous	0.7106	0.7685	-	0.9975	0.9882	-

**Table 3:** Pearson correlations among emotions in level 4a (Social and Personal focus) and level 4b (Self-protection, Anxiety-avoidance and Growth, Anxiety-free).

	**Social Focus**			**Personal Focus**		
	Positive	Negative	Ambiguous	Positive	Negative	Ambiguous
Positive	-	0.9593	0.8266	-	0.9653	0.9648
Negative	0.9593	-	0.8415	0.9653	-	0.8865
Ambiguous	0.8266	0.8415	-	0.9648	0.8865	-
	**Self-protection, Anxiety-avoidance**			**Growth, Anxiety-free**		
	Positive	Negative	Ambiguous	Positive	Negative	Ambiguous
Positive	-	0.9593	0.9634	-	0.8674	0.8731
Negative	0.9593	-	0.9769	0.8674	-	0.8434
Ambiguous	0.9634	0.9769	-	0.8731	0.8434	-

## Data Availability

Regarding our analysis and experiments, we utilized a human value dataset [11] available in (https://zenodo.org/records/10909895 accessed on 11 April 2024).
